# Melatonin Combined with Wax Treatment Enhances Tolerance to Chilling Injury in Red Bell Pepper

**DOI:** 10.3390/metabo14060330

**Published:** 2024-06-13

**Authors:** Magalí Darré, María José Zaro, Michelle Guijarro-Fuertes, Ludmila Careri, Analia Concellón

**Affiliations:** CIDCA, Centro de Investigación y Desarrollo en Criotecnología de Alimentos (CONICET-UNLP-CIC), Facultad de Ciencias Exactas, Calles 47 y 115, La Plata CP 1900, Argentina; magali.darre@agro.unlp.edu.ar (M.D.); maria.zaro@agro.unlp.edu.ar (M.J.Z.); guijarrofme@outlook.com (M.G.-F.); eilcareri@hotmail.com (L.C.)

**Keywords:** *Capsicum annuum* L., postharvest, quality, melatonin, proline, antioxidant

## Abstract

Bell peppers (*Capsicum annuum* L.) are prone to chilling injury (CI) when stored at temperatures below 7 °C. Melatonin, a natural plant regulator, plays a critical role in defending against different pre- and post-harvest abiotic stresses, including those associated with cold storage. This study aimed to assess the effects of applying exogenous melatonin alone and in combination with a commercial wax on the CI tolerance, postharvest life, and potential biomarker search of red bell peppers. In the initial experiment, the effective melatonin concentration to reduce CI effects was determined. Peppers were sprayed with either distilled water (control) or a melatonin aqueous solution (M100 = 100 μM or M500 = 500 μM) and then stored for 33 d at 4 °C, followed by 2 d at 20 °C. The M500 treatment proved to be more effective in reducing fruit CI incidence (superficial scalds) and metabolic rate, while weight loss, softening, and color were comparable to the control. A second experiment assessed the potential synergistic effects of a combined melatonin and commercial wax treatment on pepper CI and quality. Fruits were sprayed with distilled water (control), melatonin (M500), commercial wax (Wax), or the combined treatment (Wax + M500) and stored for 28 d at 4 °C, followed by 2 d at 20 °C. The Wax + M500 was the most effective in significantly reducing the incidence of fruit CI symptoms and calyx fungal infection. Furthermore, this combined treatment enhanced fruit weight loss prevention compared with individual melatonin or wax treatment. Also, Wax + M500-treated peppers exhibited notable proline accumulation, indicative of a metabolic response counteracting the cold effects, resulting in better fruit stress acclimation. This treatment also preserved the peppers’ color and antioxidant capacity. In summary, these findings highlight the suitability of applying a combined Wax + M500 treatment as a highly effective strategy to enhance the CI tolerance of peppers and extend their postharvest life.

## 1. Introduction

Red bell peppers (*Capsicum annuum* L.) are widely consumed due to their culinary versatility and nutritional value, including vitamins, minerals, and antioxidants, which promote human health and help prevent various diseases [1]. Cold storage is one of the most effective postharvest technologies applied to delay several metabolic pathways and extend the shelf-life of vegetables. However, red peppers are prone to suffer chilling injury (CI) when stored at temperatures below 7 °C, resulting in damage such as surface pitting and scalds, calyx browning, and fungal growth, which significantly reduces their shelf-life [2]. These symptoms develop rapidly and worsen when exposed to ambient conditions after being stored in cold storage [3]. So, minimized deterioration by CI is one of the main objectives to avoid quality and commercial losses.

Two general hypotheses are proposed as biochemical mechanisms of CI [4]. One is that the cell membrane is the main site of CI development, with membrane structural dysfunction and further increases in their permeability. The other one is oxidative damage at the subcellular level due to increased concentrations of reactive oxygen species (ROS), ultimately leading to CI symptoms. Then, several physiological and metabolic disorders accompany these reactions. Currently, various metabolic aspects are still being studied and specific metabolites are even being sought as rapid biomarkers of response to stress caused by cold storage. One such example is proline, a metabolite that has recently gained attention for its significant up-regulation in several fruits and vegetables exposed to cold stress. Proline is thought to act as an osmotic regulator in plant species [5]. Additionally, proline would have the potential to stabilize membrane and subcellular structures, as well as protect cells from oxidative damage during stressful periods [6,7]. 

Various postharvest control strategies have been studied worldwide to minimize the CI of horticultural crops. For peppers, several pre-storage treatments have been used to induce chilling tolerance with variable success, including heat treatment [8], methyl jasmonate [9], diphenylamine [3], and hydroxypropyl cellulose [10]. However, there is still a need for new alternatives that utilize natural compounds produced by plants themselves, which are safe for human consumption and commercially viable.

Melatonin (M; N-acetyl-5-methoxytryptamine) used to be associated with animals and humans; however, in recent years, it has been linked to a variety of vegetables, including peppers [11,12]. Melatonin acts both as a signaling molecule for enhancing the resistance of plants to biotic and abiotic stresses and as a powerful antioxidant [13]. Pre-storage melatonin treatment has been used as an effective postharvest technology to improve quality, delay deterioration, and increase the chilling tolerance of different fruits and vegetables [14]. Melatonin is believed to stabilize membranes [15] and reduce oxidative damage due to suppression of ROS [16]. Previous studies have also reported that exogenous melatonin application causes proline accumulation which then leads to improved chilling tolerance in zucchini at 200 μM [7] and tomato at 100 μM [17]. Indeed, Kong et al. [2] found that exogenous melatonin at 100 μM alleviates CI in green bell peppers, by controlling lipid metabolism and increasing antioxidant capacity. However, the effects of the treatment on the chilling tolerance of red bell peppers were not investigated. This is of interest since it is known that the sensitivity to CI is substantially different during ripening. In the immature state, peppers exhibit a green color. As they mature, they turn towards red, yellow, orange, or purple, depending on the variety. Green and breaker stages appeared to be more sensitive to CI than mature red fruit [18,19]. Therefore, optimal melatonin concentration is required, and fruit response could be different.

Pepper fruit, like other Solanaceae, also has a surface wax that protects it from dehydration [20]. However, it is a common practice to add edible wax coatings to provide an additional barrier to water diffusion and enhance the gloss of the fruit, extending post-harvest shelf-life and improving appearance [21]. Carnauba wax is a natural and safe food additive known for its high hydrophobicity and effectiveness in reducing water loss from fruits and vegetables [22]. Carnauba wax-based coatings have also been shown to reduce the rate of metabolism, delay color changes and maintain the texture of fresh produce [22,23,24]. In peppers, coating with carnauba wax effectively reduced weight loss and increased total sugar content and ascorbic acid [25]. It is even possible to enrich the carnauba wax formulation with bioactive substances to further improve its efficacy. For example, successful post-harvest treatments with carnauba wax in combination with essential oils, putrescine, and γ-aminobutyric acid have already been reported [22,26,27]. However, the potential synergistic effect of adding melatonin to a wax formulation to improve the shelf-life of peppers has not yet been investigated. 

Thus, this study aimed to assess the effects of applying exogenous melatonin alone and in combination with a commercial wax on the CI tolerance, postharvest life, and potential biomarker search of red bell peppers.

## 2. Materials and Methods

### 2.1. Plant Material and Growing Conditions

Red peppers (*Capsicum annuum* L.) were grown in La Plata, Argentina (latitude: 35°0′28.5″ S, longitude: 58°1′47″ W) in a horticultural greenhouse (2.0–3.5 m height × 10 m width × 40 m length). The region of La Plata has a soil type called Vertic Argiudoll [28], and it is characterized by a poor drainage due to the high proportion of clay from the surface; soils have a silty clay loam texture and are well supplied with organic matter (>5%), slightly acidic pH and adequate concentration of cations (conductivity < 1.5 dS m^−1^). Before transplanting, decompaction and solarization were carried out to disinfect the soil. Then, the bell pepper seedlings were transplanted in winter (July 20th) and arranged in double rows. The plants were spaced 1 m between the rows and 0.5 m between plants. Irrigation was conducted every day during spring and summer and once a week in late fall. The nutrient solution was pumped from fertilizer tanks through a drip irrigation system with one dripper per plant and a flow rate of 4 L h^−1^. Fertilization was applied by drip irrigation throughout the growing season and consisted of 80 g plant^−1^ of a commercial fertilizer containing 10 N–2.2 P–24.9 K. Fruit set was achieved through natural pollination. The fruits were harvested in summer when they were commercially ripe (14 cm long × 8 cm upper × 6 cm lower diameter). Uniform and healthy peppers were immediately transported to the laboratory. The fruits were washed and disinfected with an aqueous 200 ppm sodium hypochlorite solution. The experiences were designed using a completely randomized factorial design.

### 2.2. Melatonin Concentration

In the first experiment, fruits were sprayed with distilled water (Control), 100 µM melatonin aqueous solution (M100), or 500 µM melatonin aqueous solution (M500). Tween 20 at 0.1% was added as a surfactant to all treatments. Then, the fruits were air-dried and kept in pairs on polyethylene terephthalate (PET) trays covered with perforated PVC film. To replicate commercial conditions, they were kept for up to 33 days at 4 °C and then transferred for 2 days at 20 °C. Fruit damage index (pitting and superficial scalding), weight loss, respiration rate, color, and firmness were used to determine the optimal melatonin concentration. Twenty fruits were examined for each treatment and storage time, and the experiment was carried out two times.

### 2.3. Combined Treatment of Melatonin and Commercial Wax

In a second experiment, edible wax (Emulcol TM^®^, Wassington-Agro, Buenos Aires, Argentina), and formulated based on carnauba wax (8%) shellac (4%) was diluted to 30% with distilled water and then combined with the melatonin concentration chosen in the earlier assay. There were four treatments: Control, Wax, M500, and Wax + M500. The treatments were carried out in the same way as in the first experiment. To replicate commercial conditions, the fruits were kept for up to 28 days at 4 °C and then transferred for 2 days at 20 °C. Twenty fruits were examined for each treatment and storage time, and the experiment was carried out three times.

### 2.4. Incidence of Chilling Injury (CI)

Red peppers were unmarketable when they reached 40% of the fruit or calyx area affected with CI symptoms. Then, the incidence of CI in the fruit (pitting and scalding) or calyx (browning, dehydration, and fungal attack) was calculated based on the percentage of damaged areas of fruits or calyxes affected by symptoms as follows. Twenty fruits were examined for each treatment and storage time.

CI Incidence (%) = (Number of fruits with~40% affected area/total number of fruits) × 100

Calyx deterioration incidence (%) = (number of fruits with calyx~40% affected area/total number of fruits) × 100

### 2.5. Weight Loss

Each fruit was weighed at 0 d and subsequent sampling days. Twenty fruits were examined for each treatment and storage time, and the results were expressed as a percentage of weight loss compared to the initial weight.

### 2.6. Respiration Rate

Two fresh fruits were weighed, placed in a 3 L sealed glass jar, and incubated at 25 °C for 15 min. The CO_2_ produced was measured to determine the respiration rate, using an IR analyzer (Alnor, CompuFlow Model 8650, Shoreview, MN, USA) [29]. The results were presented as mg of CO_2_ kg^−1^ h^−1^. Three biological replicates were conducted for each treatment and storage time. 

### 2.7. Surface Color

The external color of the fruits was measured using a chroma meter (Minolta, Model CR-400, Osaka, Japan) [29]. According to the CIE color model, the L*, a*, and b* values were determined. The a* value represents redness or greenness (−a = green, +a = red), the b* value represents blueness or yellowness (−b = blue, +b = yellow), and the L* value denotes lightness (0 = black, 100 = white). The values of a* and b* were transformed into hue angle (Hue, arctan (b/a)). Ten fruits were examined for each treatment and storage time, with three measurements per fruit.

### 2.8. Firmness

Fruit firmness was measured utilizing 1 × 3 cm strips that were horizontally laid out and supported at both ends by platforms. A normal force was applied by a tooth-shaped probe to the center of the pepper strip (the side of the cuticle) in a bending test as described by Rodoni et al. [29]. The maximum force (N) needed to break the pepper strips was measured using a texture analyzer (TA.XT2. Stable Microsystems, Godalming, UK). Ten fruits were examined for each treatment and storage time, with three measurements or strips per fruit.

### 2.9. Trolox Equivalent Antioxidant Capacity (TEAC)

The TEAC was determined using the ABTS method described by Rodoni et al. [29]. The antioxidants were extracted with 3 g of frozen, ground-up tissue and 10 mL of ethanol. The mixture was centrifuged at 10,000× *g*, and the supernatant was saved. To measure, 50 µL of extract were added to 1 mL of ABTS^·+^ radical solution (absorbance 0.700 ± 0.03 at 734 nm). After 6 min, the absorbance at 734 nm was evaluated using a UV/visible spectrophotometer (UV-Mini Model 1240, Shimadzu, Kyoto, Japan). Two independent extractions were performed for each treatment and storage time, and each sample was evaluated in triplicate. Trolox was used as the antioxidant standard and the results were expressed as TEAC in mg kg^−1^ on a fresh tissue. 

### 2.10. Proline Content

The proline content was assessed using the procedure outlined by Abraham et al. [30]. So, 8 mL of 3% sulfosalicylic acid was used to homogenize 3 g of frozen and crushed fruit tissue. Then, 1 mL of the extract was mixed with 1 mL acid ninhydrin (diluted in glacial acetic acid and 6 M phosphoric acid) in a test tube and boiled at 100 °C for 1 h in a water bath. The mixture was then quickly cooled for 10 min in an ice bath. Thereafter, 2 mL of toluene was added and the absorbance of the organic phase at 520 nm was measured using a UV/visible spectrophotometer (UV-Mini Model 1240, Shimadzu, Japan). Proline (Sigma, St. Luis, MO, USA) was used as a standard. Two independent extractions were performed for each treatment and storage time, and each sample was evaluated in triplicate. The results were expressed as μg kg^−1^ of fresh tissue.

### 2.11. Statistical Analysis

The experiences were designed using a factorial design. The first experiment included three treatments (Control, M100, and M500), with observations at 0, 21 + 2, and 33 + 2 days. In the second experiment, four treatments (Control, M500, Wax, and Wax + M500) were tested and evaluated at 0, 14 + 2, 21 + 2, and 28 + 2 days. All data were analyzed with the Infostat program using ANOVA. Fisher’s test was used to evaluate least significant differences (LSD) at *p* < 0.05.

## 3. Results and Discussion

### 3.1. Melatonin Effectiveness and Concentration Selection

**Figure 1:**
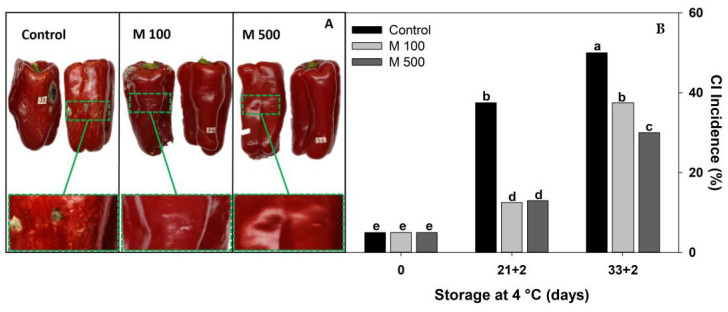
Selection of melatonin treatment at different concentrations: 0 µM (Control), 100 µM (M100), and 500 µM (M500). (**A**) Fruit general appearance at 33 + 2 days, and a magnification of the peel symptom delimited by the box. (**B**) Chilling injury Incidence of red bell pepper fruits during storage at 4 °C for 33 days and then being transferred at room temperature for 2 days (33 + 2). Different letters indicate significant differences according to Fisher’s LSD test (*p* < 0.05).

Pitting and browning lesions are usually the initial CI symptoms in bell peppers [2,9,10]. Also, in long periods of storage, the damaged tissue is susceptible to fungal attack. Melatonin treatments were effective in reducing the severity of pitting and scalds in fruits stored at chilling temperature for 33 days and then transferred for 2 days at 20 °C (33 + 2) (Figure 1A). The M100 treatment showed an intermediate degree of pitting, whereas M500 exhibited the least pitting and managed to inhibit the fungal attack that is observed in control fruits with an advanced degree of lesion in their tissue. Probably, melatonin acted as a direct and powerful antioxidant, as previously reported [13]. Both concentrations, M100 and M500, produced a substantial and comparable 66% decrease in the severity of CI incidence compared to the control after 21 + 2 d (Figure 1B). M100 reduced fruit CI by 25% at the end of storage (33 + 2 d), while M500 was significantly more successful at this time, achieving a 40% reduction in CI incidence compared to untreated fruit (Figure 1B). These findings contrast with those reported by Kong et al. [2] who, working with green peppers, identified M100 as the most effective treatment for delaying the onset and severity of CI symptoms. These differences could be attributed to the heightened susceptibility of green peppers to CI, which showed significant deterioration after 15 and 20 days. In contrast, red peppers were more tolerant to CI since symptoms initially appeared after 21 + 2 d, and fruit remained marketable for 33 + 2 d (Figure 1B). Thus, to control CI in red peppers over long periods, a high concentration of melatonin was required. The optimal concentration identified here was 500 µM melatonin, which can be considered intermediate. It is worth noting that concentrations ranging from 50 to 1000 µM have been reported for effective postharvest treatments in various fruits and vegetables [14].

**Figure 2:**
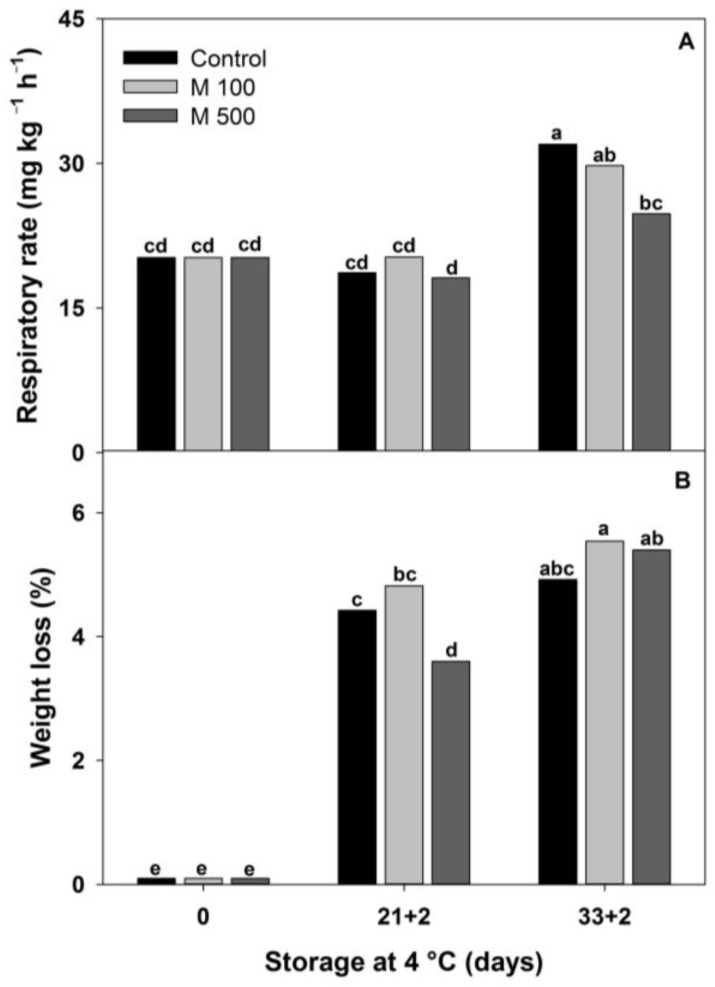
Selection of melatonin treatment at different concentrations: 0 µM (Control), 100 µM (M100), and 500 µM (M500). (**A**) Respiration rate. (**B**) Weight loss of red bell pepper fruits during storage at 4 °C for 33 days and then being transferred at room temperature for 2 days (33 + 2). Different letters indicate significant differences according to Fisher’s LSD test (*p* < 0.05).

A high respiration rate is one of the main causes of deterioration and senescence in postharvest fruit and vegetables [18]. A good postharvest treatment needs to be effective in reducing this occurrence. The respiration rate of red peppers was not affected by melatonin treatment until 21 + 2 d (Figure 2A). However, at 33 + 2 d, M500 lowered the respiration rate compared to the control and M100 by 22% and 17%, respectively. This is important since fruit respiration is the physiological parameter that usually correlates well with fresh fruit perishability or integral metabolic activity [28]. Usually, postharvest treatments could alleviate CI by maintaining the membrane integrity, allowing proper ATP production during respiration [4]. So, a lower respiration rate would indicate the best cell and tissue integrity.

Weight loss is a critical factor that has been associated with shriveling and CI symptoms in several fruits and vegetables [7,31]. Weight loss increased with storage time in all fruits. Meanwhile, at 21 + 2 d, the M500 treatment reached a level of 3.6%, which was significantly lower than the values of 4.4% and 4.8% from the control and M100, respectively (Figure 2B). However, during long periods of storage, M500’s efficacy to prevent pepper fruit dehydration was reduced and finished with a comparable weight loss to the control and M100 fruits (~5.2%) (Figure 2B). Here, the melatonin treatment was not forceful enough to control dehydration during the long storage times required for red peppers. In general, weight loss was effectively controlled in other melatonin-treated fruits; however, this response could be influenced by fruit species, storage temperature, and melatonin concentration [14]. In zucchini, a 1.63-fold less weight loss was found in fruit treated with melatonin 200 µM after 15 d at 5 °C [7], while in mango treated with melatonin 1000 µM, the weight loss was delayed until ~1.10-fold fewer values than control after 27 d at 13 °C [31].

**Figure 3:**
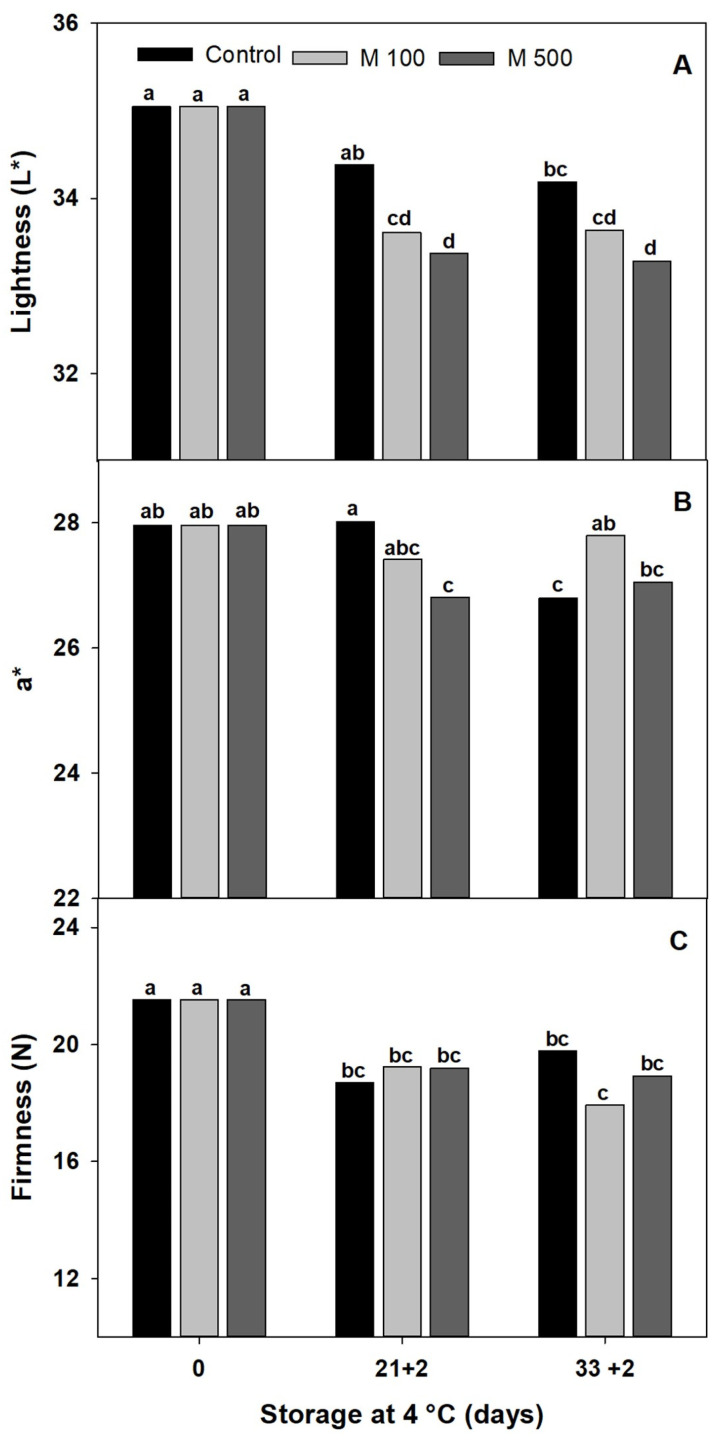
Selection of melatonin treatment at different concentrations: 0 µM (Control), 100 µM (M100), and 500 µM (M500). (**A**) Luminosity, or L*. (**B**) a*. (**C**) Firmness of red bell pepper fruits during storage at 4 °C for 33 days and then being transferred at room temperature for 2 days (33 + 2). Different letters indicate significant differences according to Fisher’s LSD test (*p* < 0.05).

The external color is an important factor in conserving the freshness and visual quality of red peppers stored under cold storage [32]. However, it could be affected by the advancement of ripening, senescence, or CI. The color usually changes during postharvest storage, but alterations are more relevant when peppers are in breaker stage than in green or red fruit [18,19]. When the color is full, green or red, the changes are minor. Here, the red color of treated peppers slightly darkened with storage time, shown by the lowest L* and a* values (Figure 3A,B), denoting minor advances in maturity or senescence. However, there were no appreciable differences between the M100 and M500 treatments after 21 + 2 and 33 + 2 d.

Fruit firmness and freshness are important quality factors during pepper postharvest [15]. The firmness of peppers showed a slight decline (15%) during 33 + 2 d of storage, but no appreciable differences were found between the M100 and M500 treatments or even compared to the control at each sampling date (Figure 3C). Fruit firmness decreases during postharvest as a natural process of cell wall disassembly due to the depolymerization and solubilization of pectin and cellulosic material [7]. Some products with advanced CI symptoms showed an accelerated reduction of turgor due to the increment in softening enzyme activity [4]. Here, the softening of red peppers was comparable between control and treated fruits, indicating that cell wall material was not greatly affected by low temperatures. 

Overall, the results show that both M100 and M500 treatments delayed the onset of chilling injury in red bell pepper during storage at 4 °C. In addition, M500 significantly reduced the respiration rate, while it did not affect the color and firmness of the fruit. M500 also controlled weight loss, but only until the middle of the storage period. Therefore, M500 was the best concentration of the treatment, but there was still a need to achieve better control of weight loss throughout the entire storage period.

### 3.2. Effect of Melatonin and Wax Used Alone or as Combined Treatment on Red Pepper CI Incidence

**Figure 4:**
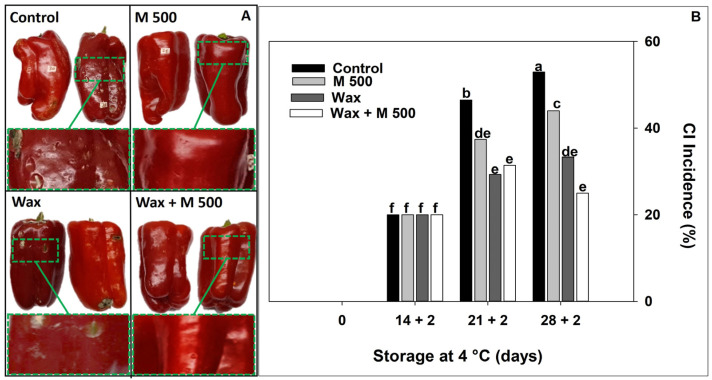
Effect of treatments: 0 µM (Control), 500 µM melatonin (M500), wax, and combined (wax + M500). (**A**) Fruit general appearance at 28 + 2 days, and a magnification of the peel symptom delimited by the box. (**B**) Chilling injury incidence of red bell pepper fruits during storage at 4 °C for 28 days and then being transferred at room temperature for 2 days (28 + 2). Different letters indicate significant differences according to Fisher’s LSD test (*p* < 0.05).

To enhance the efficacy of melatonin treatment throughout the entire storage period, a combined treatment with commercial wax (based in carnauba) was assayed. Commercial wax has many benefits when used in the post-harvest of fruits and vegetables, including being a barrier against oxygen, moisture, and solute losses [24]. Furthermore, it imparts a nearly transparent and shiny surface [33]. It also has many practical uses such as faster drying times after washing, which was confirmed in our tests. Additionally, coating based on galactomannan and carnauba wax protects guava fruit from CI and maintains its firmness [34]. These coatings can also be formulated with compounds which had antioxidant or antimicrobial properties [24]. Here, we assessed the effect of Wax treatment (Emulcol TM^®^) added with melatonin (M500) on both pepper CI incidence and quality. All treatments decreased pitting and lesions compared to the control fruits at long storage times (28 + 2 days) (Figure 4A). Moreover, the use of melatonin continued to show fewer pitting symptoms and inhibition of fungal attack in both treatments: M500 and Wax + M500. An earlier instance of time was studied (14 + 2 d), to evaluate whether mild symptoms appeared, or if biochemical responses were generated before the visual symptom. CI symptoms, such as pitting and scalds, appeared at 21 + 2 d progressing with different degrees of severity depending on the treatment (Figure 4A,B). At 21 + 2 d, fruit treated with Wax, M500, and Wax + M500 exhibited good appearance and lower CI incidence compared to the control fruit. At the end of storage (28 + 2 d), Wax and Wax + M500-treated peppers showed the lowest CI incidence, although with differences between them that did not end up being statistically significant. Nevertheless, it is noteworthy that Wax + M500-treated fruit showed 50% fewer CI symptoms than the control, proving also to be more effective than M500 alone. Therefore, the use of a wax coating could have reduced the permeability or gas exchange with the surrounding environment of the peel tissue and therefore slowed down the production of ROS [22,27]. Due to this, treatment with Wax probably also reduced CI. Similar results were found when treatments of putrescine and carnauba wax were applied to mitigate pomegranate CI [26]. Finally, this effect could be added to the already mentioned properties of melatonin, resulting in a synergistic effect between them that ends up increasing the reduction of CI of red pepper.

**Figure 5:**
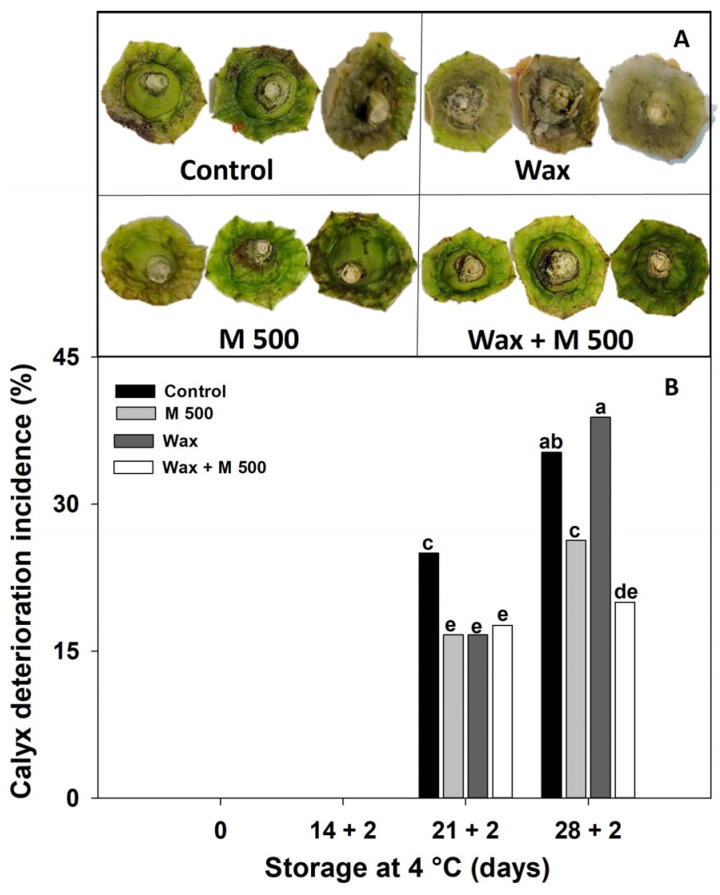
Effect of treatments: 0 µM (Control), 500 µM melatonin (M500), wax, and combined (wax + M500). (**A**) Calyx appearance at 28 + 2 days. (**B**) Incidence of calyx deterioration of red bell pepper fruits during storage at 4 °C for 28 days and then being transferred at room temperature for 2 days (28 + 2). Different letters indicate significant differences according to Fisher’s LSD test (*p* < 0.05).

When peppers are stored for long periods, their calyxes also deteriorate at a similar rate to the fruit. Diverse strategies to prevent calyx decay, such as hot water or UV-C treatments, were previously assayed [29,35]. Here, all treatments applied were effective in controlling CI symptoms and preserving pepper calyx appearance until 21 + 2 d reducing browning, dehydration, and fungal attack compared to the control fruit (Figure 5B). Surprisingly, at longer storage times (28 + 2 d), the Wax treatment had a detrimental effect, causing increased calyx deterioration and fungal development even similar to the control (Figure 5A,B). This occurrence negates the possibility of considering this wax treatment for long-term postharvest conservation of red pepper, because the quality and safety of the calyx is essential for its correct marketing. In contrast, the M500 treatment resulted in a decrease in the percentage of fruits exhibiting calyx deterioration and fungal attack (Figure 5A,B). Additionally, the treatment combination Wax + M500 proved to be the most successful in delaying the onset of general deterioration and fungal attack in the calyx. These results suggest that melatonin may have a direct impact on microorganisms, like the effects proposed for peaches [16] and mangoes [31]. Melatonin had proven immune-modulatory and anti-inflammatory effects, indicating that it can inhibit bacterial, viral, and parasitic infections [12]. Therefore, the M500 treatment reduced decay and its effect was enhanced when it was combined with Wax + M500, resulting in this combination being the most effective treatment.

**Figure 6:**
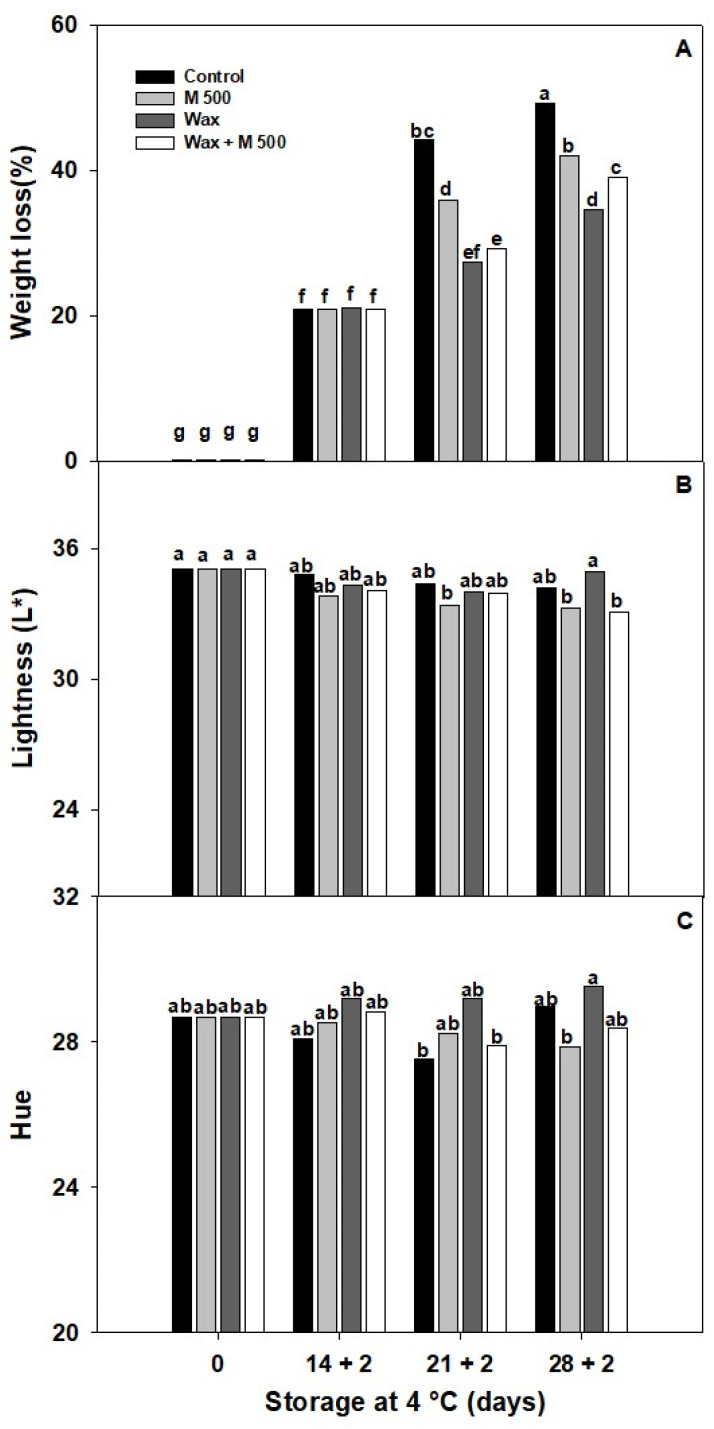
Effect of treatments: 0 µM (Control), 500 µM melatonin (M500), wax, and combined (wax + M500). (**A**) Weight loss, (**B**) Lightness (L*), (**C**) Hue angle of red bell pepper fruits during storage at 4 °C for 28 days and then being transferred at room temperature for 2 days (28 + 2). Different letters indicate significant differences according to Fisher’s LSD test (*p* < 0.05).

Prolonged storage resulted in increased weight loss in both the control and treated peppers (Figure 6A). According to the first experiment, M500 showed a significant reduction related to the control by 19% and 15% at 21 + 2 d and 28 + 2 d, respectively. Notably, the Wax and Wax + M500 treatments showed better outcomes, resulting at 21 + 2 d in ~35% lower than the control, while at the end of storage, 28 + 2 d, both treatments showed some % differences. Wax treatment was the most effective in reducing fruit dehydration by 30% compared to the control, while in combination with melatonin (Wax + M500) the reduction was 20%, altering their effectiveness (Figure 6A). The findings indicate that melatonin may influence the structure and coating capacity of the wax. While the mechanical and barrier properties of active coatings represent a significant area of postharvest research, the combination of wax and melatonin remains unexplored. Therefore, these results need further and more comprehensive research.

The fruits’ lightness (L*) remained within the range of values from 32 to 35 (Figure 6B) and Hue values between 27 and 29 (Figure 6C) throughout the storage period, and no major changes were seen between treatments. This finding makes it possible to confirm minor changes in the progress of fruit ripening or senescence and, on the other hand, that the gloss of the fruit remained unchanged. Similar results were achieved by bioactive coatings with wax, pullulan, or chitosan, whose results were also effective in extending the shelf-life of apples and red bell peppers without changing their appearance [36,37].

In summary and as expected, the commercial Wax was highly effective in reducing the red pepper weight loss, even at long storage times, but the lack of control of calyx fungal decay was an undesirable result that negatively affected their functionality and marketable condition. This would not allow the use of wax as an alternative treatment in post-harvest. On the other hand, pretreatment with M500 prevented fruit CI, calyx deterioration, and decay but was not effective in reducing weight loss. Nevertheless, a synergist effect and best results were observed when the combination of Wax + M500 was used, since this treatment reduced the CI symptoms, calyx deterioration, and weight loss even during prolonged storage periods, without affecting the fruit color. Therefore, Wax + M500 can not only alleviate the chilling tolerance in red peppers but also can maintain the fruit quality.

### 3.3. Effect of Melatonin and Wax Combined Treatment on Chilled Red Pepper TEAC and Proline Content

**Figure 7:**
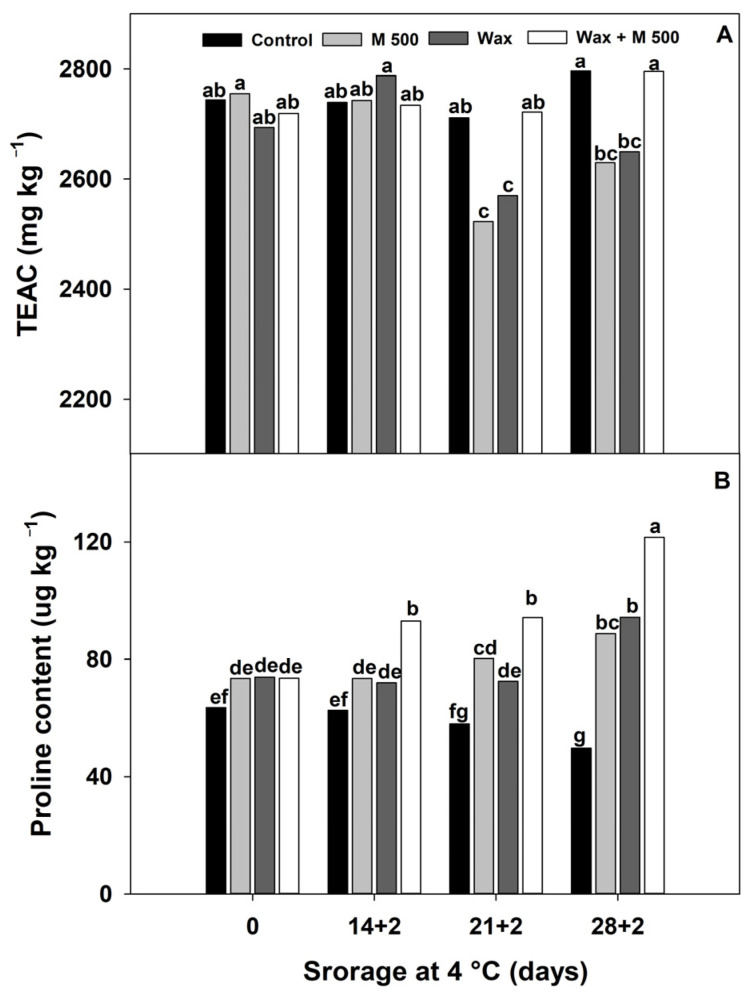
Effect of treatments: 0 µM (Control), 500 µM melatonin (M500), wax, and combined (wax + M500). (**A**) TEAC (Trolox equivalent antioxidant capacity) and (**B**) proline content of red bell pepper fruits during storage at 4 °C for 28 days and then being transferred at room temperature for 2 days (28 + 2). Different letters indicate significant differences according to Fisher’s LSD test (*p* < 0.05).

Red peppers are a good source of hydrophilic antioxidants such as phenolic compounds and vitamin C, which are very good ROS scavengers [1,2]. In this study, the antioxidant capacity determined as TEAC of M500 and Wax-treated fruit slightly decreased after 21 + 2 d and remained low until 28 + 2 d (Figure 7A). However, Wax + M500 treated red bell peppers did not significantly change during storage. This lack of response from peppers could be due to several possibilities. One of them could be a similar consumption and production rate of hydrophilic antioxidants such as phenolic compounds. Another possibility could be related to the fact that the less oxygen is exchanged through the Wax and therefore, less oxidative reactions take place. In addition, melatonin activates metabolic pathways involved in maintaining redox status and scavenging ROS [13,14]. When treated with Wax + M500, both could synergistically contribute to the non-alteration of TEAC. Wang et al. [12] reviewed the effects of melatonin in postharvest and mentioned that antioxidants usually increase in fruits and vegetables, but effects on red bell pepper were not mentioned. These authors summarized that melatonin treatment also increases the activity of enzymes that detoxify ROS, which mitigates lipid peroxidation and ultimately contributes to better membrane integrity and maintains the integral postharvest quality of produce. Therefore, the measurement of TEAC would not be a good indicator of the stress response in red bell peppers, and further studies on the activities of antioxidant enzymes need to be conducted. Other authors also found no differences in TEAC effects in red peppers stored at chilling temperatures and pretreated with UVC [29,32] or immersed in hot water at 53 °C [35].

In recent years, various studies have found that the proline amino acid is an important metabolite that contributes to the adaptability of fruit and vegetables to biotic and abiotic stresses [38]. Nevertheless, the efficacy of proline against cold stress in red peppers remains unexplored to date. Proline is biosynthesized from Arginine, an amino acid that provides nitrogen for several metabolic pathways (included polyamines like putrescine, spermidine, and spermine) and also acts as a supplier of carbon skeleton [14]. In biological cells, exogenous melatonin promotes the arginine pathway [14], and the activation or suppression of determined enzyme activity could finally redirect this amino acid to produce certain metabolites as essential signaling molecules. Here, we decided to evaluate one of these molecules and the proline content was measured in control and treated red peppers. The proline content of the control peppers remained constant until 21 + 2 d (Figure 7B), while proline from Wax and M500 peppers were slightly higher than control and remained constant until 21 + 2 d. Then, the proline content of untreated peppers slightly decreased at 28 + 2 d, but increased 50% in Wax and M500 peppers compared to the control. It is noteworthy that the Wax + M500 treatment significantly induced proline accumulation even earlier (14 + 2 d) and it peaked at 28 + 2 d, with an increase of 60% compared to the control and 22–26% regarding the Wax and M500 treatments, respectively. Several studies have proposed proline as a signal molecule in various types of stress [14]. In accordance, pre-treatment with melatonin also caused increments of proline in chilled green pepper [2], peach [15], cucumber [6], zucchini [7], and sapota [39]. Some studies found that the exogenous melatonin stimulated the activity of biosynthetic enzymes of proline and suppressed its degradation [6,7,14]. These studies suggest that proline could act as an osmotic regulator, playing a crucial role in enhancing cellular osmolarity. Also, it can act as an ROS scavenger contributing to increase stress tolerance, stabilizing membrane and subcellular structures, and finally favoring cellular integrity in cold-stored produce [6,14]. 

In brief, our findings suggest that the Wax and M500 treatments had an impact on proline accumulation in red bell peppers as the abiotic stress effect added to low temperature stress. In this sense, when both treatments were combined as Wax + M500, the stress effect would be cumulative resulting in earliest and highest levels of proline achieved. It was interesting to note that the proline level increased at 14 + 2 d in Wax + M500, the day at which red pepper symptoms (pitting and browning lesions) are absent. Thus, proline could be considered as a rapid biomarker of red peppers in response to cold stress, which enhances fruit acclimation. Further studies are needed to establish the extent at the metabolic level of the benefits achieved by this biomarker increase.

## 4. Conclusions

The results of this study show that red bell peppers had a significantly longer shelf-life and less chilling injury when 500 µM melatonin (M500) was applied as a pre-storage treatment. Treatment with M500 effectively reduced the incidence of chilling injury and the respiration rate, while color and firmness were comparable to those of the control peppers. In addition, weight loss was not effectively controlled until the end of the long storage period. In contrast, when melatonin was combined with a commercial wax coating (Wax + M500), it had a synergistic effect that reduced weight loss, calyx decay incidence, and chilling injury symptoms without altering the visual appearance of the fruit or TEAC. This combined treatment also promoted proline accumulation in response to the effects of cold stress and improved fruit acclimatization. Overall, this study shows that pretreatment with a combination of Wax + M500 is a natural and effective method to improve the cold tolerance of red bell peppers and extend their postharvest life.

## Data Availability

Data presented in this study are available on request from the corresponding author due to privacy.
